# Synthesis of Certain Pyrimidine Derivatives as Antimicrobial Agents and Anti-Inflammatory Agents

**DOI:** 10.3390/molecules15031882

**Published:** 2010-03-15

**Authors:** Mosaad S. Mohamed, Samir M. Awad, Amira Ibrahim Sayed

**Affiliations:** Pharmaceutical Organic Chemistry Department, Faculty of Pharmacy, Helwan University, Helwan, Egypt

**Keywords:** pyrimidine, 6-amino-2-thioxo-*1H*-pyrimidine-4-one, antimicrobial, anti-inflammatory

## Abstract

A variety of novel bicyclic and tricyclic pyrimidine derivatives was obtained via reaction of 6-amino-2-thioxo-*1H*-pyrimidine-4-one (**1**) with a different reagents. The antimicrobial and anti-inflammatory activities of some of the synthesized compounds were tested.

## 1. Introduction

Pyrimidines exhibit a range of pharmacological activity such as antibacterial [1,2,3], antifungal [4,5], anticancer [6,7], anti-inflammatory [8,9] and cardioprotective effects [10]. These observations led us to attempt the synthesis of some new pyrimidine derivatives using 6-amino-2-thioxo-2,3-dihydro-1*H*-pyrimidine-4-one (**1**) as starting material.

## 2. Results and Discussion

The requisite starting material **1** was prepared by the condensation of thiourea with ethylcyanoacetate in sodium ethoxide according to the known procedure [11]. This compound was previously used in the synthesis of several pyrimidines, for example 6-{[1-phenyl or (4-methoxyphenyl)-methylidene]-amino}-2-thioxo-2,3-dihydro-1*H*-pyrimidin-4-one [12] and 7-amino-5-(4-chlorophenyl)- or (4-methoxyphenyl)-4-oxo-2-thioxo-1,2,3,4-tetrahydropyrido[2,3-d]pyrimidine-6-carbonitrile [13]. 

**Scheme 1:**
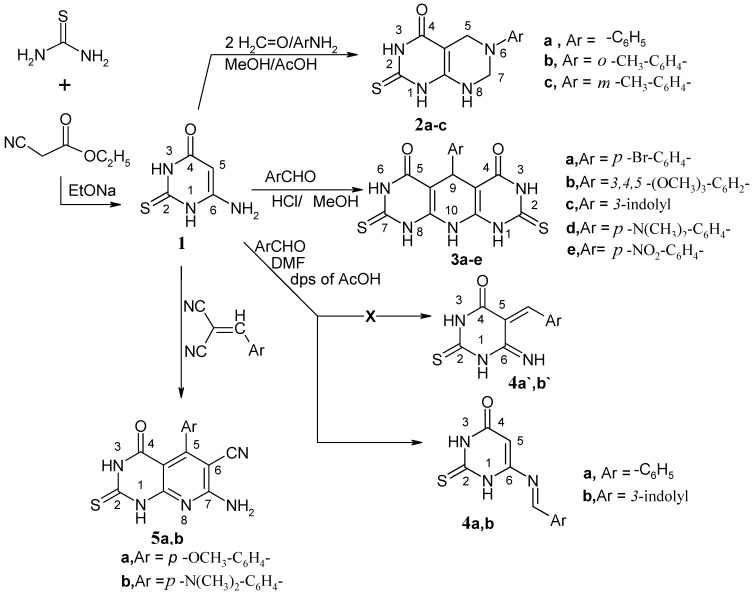
Synthetic pathway of compounds (1–5).

Stirring of **1** with primary amines and excess formalin in methanol in presence of acetic acid afforded 6-aryl-2-thioxo-2,3,5,6,7,8-hexahydro-*1H*-pyrimido[4,5-*d*]pyrimidine-4-ones **2a**-**c** (Scheme 1) and not the 8-hydroxymethyl derivatives of **2a**-**c**, as reported earlier [11], a conclusion supported by the absence of signals corresponding to an 8-CH_2_OH group in the ^1^H-NMR spectra of the products **2a**-**c**. 

Reaction of **1** with aromatic aldehydes in methanol containing few drops of hydrochloric acid (pH = 5-6) led to the formation of 9-substituted-1,3,6,8,9,10-hexahydro-2,7-dithioxopyrido[2,3-*d*:6,5 *d*`] dipyrimidine-4,6-diones **3a**-**e**. In this reaction, the polar and acidic condition of solvent make the reaction occur through the formation of a 6-imino group that leads to higher nucleophilicity of C-5 whereby attack occurs at the aldehyde carbonyl group (Scheme 1).

On the other hand, when we used a non-polar and slightly acidic solvent (DMF and few drops of acetic acid, pH = 6.5–7), the imino group wasn’t formed so the reaction proceeds via nucleophilic attack of the amino group on the aldehyde carbonyl group leading to the 6-{[1-aryl-methylidene]-amino}-2-thioxo-2,3-dihydro-*1H*-pyrimidine-4-one condensation products **4a**,**b** and not **4a′**,**b′**, as reported earlier [12]. The formation of the isomeric imino compounds **4a′,b′** is not plausible because the amino group is much more nucleophilic than the CH at position 5 of 6-amino-2-thioxo-*1H*-pyrimidine-4-one (**1**); in addition the ^1^H-NMR showed one proton signal at δ = 5.26 ppm for the pyrimidine H-5 and one proton signal at δ = 7.84 ppm for the azamethine proton (N=CH), confirming fortmation of the structures **4a**,**b**.

Refluxing compound **1** with arylidene malononitriles in absolute ethanol containing a few drops of triethylamine afforded 7-amino-5-aryl-4-oxo-2-thioxo-1,2,3,4- tetrahydro -pyrido[2,3-*d*] pyrimidine-6-carbonitriles **5a**,**b** (Scheme 1). The structures of all new synthesized compounds were confirmed by IR, ^1^H-NMR, MS and elemental analysis results.

### 2.1. Antimicrobial activity

The *in vitro* antimicrobial activity of the newly synthesized compounds against *Escherichia coli* (ATCC 25922) as an example of Gram negative bacteria, *Bacillus subtilis* (ATCC-6633) as a Gram positive bacteria and *Candida albicans* (ATCC-10231) as a fungus was investigated using the disk-diffusion method [14] at the Department of Microbiology and Immunology, Faculty of Pharmacy, Helwan University, Helwan, Egypt. Ampicillin was used as an antibacterial standard and nystatin as a standard antifungal agent. The results are given in Table 1.

### 2.2. Anti-inflammatory activity *[15]*

**Figure 1:**
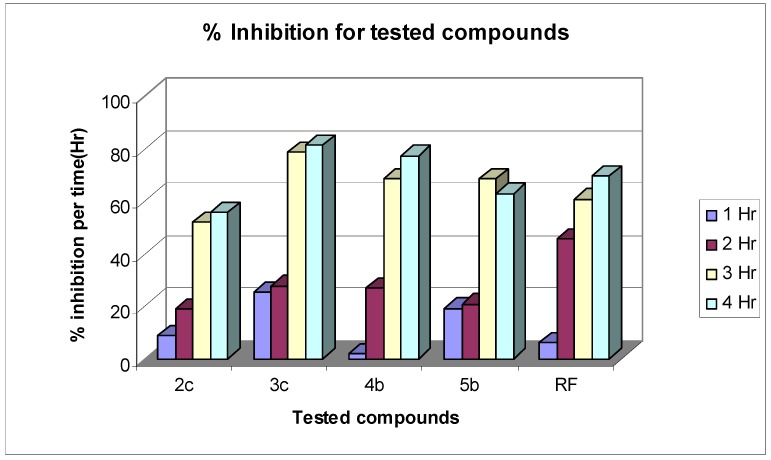
Anti-inflammatory test result.

The anti-inflammatory activity of compounds **2c**, **3c**, **4b, 5b** was investigated in comparison with ibuprofen as standard anti-inflammatory agent (Table 2, Figure 1). 

The carrageenan-induced rat paw oedema assay was carried out using a modified Winter’s method as a preliminary screening test [16]. The rats (in groups of five animals weighing 120–170 g, young adult male Sprague–Dawley) were housed in a controlled environment and provided with standard rodent chow and water for 24 h before a dose of the test compounds (50 and 70 mg/kg sc) was administered. One hour later, the volume of the right hind paw was measured, and 0.05 mL of a 1% suspension of carrageenan in sterile pyrogen-free 0.9% NaCl solution was injected subcutaneously into planter aponeurosis of the hind paw. One hour after the injection of carrageenan, the paw volume was again measured by a water pletysmometer. The mean increase of paw volume at each time interval was compared with that of control group (five rats treated with carrageenan, but without test compounds) at the same time intervals. The percentage inhibition values were calculated according to the formula: % Anti-inflammatory activity = (1- Rt/Rc)*100 (Rt = result of tested group; Rc = result of control group). All experiments involving animals were carried out using protocols approved by the Animal Committee, University of Barcelona (Spain). Animal care was in compliance with Generalitat of Catalunya regulations on protection of animals used for experimental and other scientific purposes.

## 3. Experimental

### 3.1. General

All melting points are uncorrected. Elemental analyses were carried out in the Microanalytical Center, Cairo University, Giza, Egypt. IR spectra (KBr) were recorded on Pye Unicam SP 1200 Spectrophotometer. ^1^H-NMR spectra were recorded in DMSO-d_6_ on a 90 MHz Varian NMR spectrometer using TMS as an internal standard and chemical shifts are expressed as δ ppm units. The mass spectra were determined with HP model MS-5988 at electron energy 70 eV. The homogeneity of all compounds synthesized was checked by TLC on 2.0 cm × 6.0 cm aluminum sheets coated to a thickness of 0.25 µm with silica gel 60 containing a fluorescent indicator. Characterization data of the various compounds prepared are given in Table 3 and Table 4. 

### 3.2. 6-Aryl-2-thioxo-2,3,5,6,7,8-hexahydro-1H-pyrimido[4,5-d]pyrimidine-4-ones ***2a**-**c***

A mixture of 6-amino-2-thioxo-2,3-dihydro-*1H*-pyrimidine-4-one (**1**, 0.002 mol) which was prepared as described in the literature [11] in methanol (20 mL) and acetic acid (2 mL) was heated to 40 °C and then primary aromatic amines (0.002 mol) in methanol (5 mL) and formalin (40%, 0.004 mol) were added dropwise with stirring until a clear solution was obtained. The product was filtered, washed with ethanol and dried to give compounds **2a**-**c**.

### 3.3. 9-Substituted-1,3,6,8,9,10-hexahydro-2,7-dithioxopyrido[2,3-d:6,5d′]dipyrimidine-4,6-diones ***3a**-**e***

To 6-amino-2-thioxo-2,3-dihydro-*1H*-pyrimidine-4-one (**1**, 0.002 mol) in methanol (10 mL) and concentrated hydrochloric acid (0.4 mL), the appropriate aromatic aldehyde (0.001 mol) was added and stirred at room temperature for 4.0 h, the product was collected by filtration and recrystallized from aqueous acetic acid.

### 3.4. 6-{[1-Arylmethylidene]amino}-2-thioxo-2,3-dihydro-1H-pyrimidine-4-ones ***4a**,**b***

To a solution of 6-amino-2-thioxo-2,3-dihydro-*1H*-pyrimidine-4-one (**1**, 0.01 mol) in DMF (30 mL), an equivalent amount of an aromatic aldehyde (0.01 mol) and few drops of acetic acid were added, and the reaction mixture was heated under reflux for 4 h. The reaction mixture was allowed to cool and poured on to ice/water, the product was filtered and crystallized from ethanol/dioxane mixture (1:1).

### 3.5. 7-Amino-5-aryl-4-oxo-2-thioxo-1,2,3,4- tetrahydropyrido[2,3-d] pyrimidine-6-carbonitriles ***5a**,**b***

A mixture of equimolar amounts of 6-amino-2-thioxo-2,3-dihydro-*1H*-pyrimidine-4-one (**1**) and the appropriate arylidene malononitrile (0.002 mol) in absolute ethanol (10 mL) containing triethylamine (5 drops) was heated at reflux for 3–6 h. The reaction mixture was concentrated and cooled. The solid obtained was filtered off, washed with ethanol and crystallized from DMF/ethanol (2:1).

## 4. Conclusions

The results show that compounds **2a**, **3a**,**b**,**c**, **4a**,**b** are active as antibacterials. Compound **3c** is active as an antifungal agent, while the other compounds are inactive toward the tested organisms. From the obtained results, we note that cyclisation at the 5,6-positions of 6-amino-2-thioxo-2,3-dihydro-1*H*-pyrimidine-4-one (**1**) may abolish or decrease the antimicrobial activity, as suggested by the activities of the bicyclic pyrimido[4,5-d]pyrimidine-4-one (**2a**-**c**) and pyrido[2,3-d] pyrimidine-6-carbonitrile (**5a**,**b**) and the tricyclic pyrido[2,3-d:6,5 d`] dipyrimidine (**3a**-**e**) systems. 

Concerning the anti-inflammatory activity, analysis of the results obtained using one-way analysis of variance (ANOVA) shows that there is a significant difference of all compounds from control at 3 h and 4 h At 3 h, compounds **3c**, **4b** and **5b** show higher activity than ibuprofen, while compound **2c** shows less activity. At 4 h, compounds **3c**, **4b** show higher activity than ibuprofen while compound **5b** joined **2c** in showing less activity. This lesser activity of compounds **2c**, **5b** may be due to cyclisation of the 5,6-positions of 6-amino-2-thioxo-2,3-dihydro-1*H*-pyrimidine-4-one (**1**) and the higher activity of compounds **3c**, **4b** may be due to the presence of an indolyl group as substituent. 

## Figures and Tables

**Table 1:** Antimicrobial activity of the synthesized compounds expressed as size of the inhibition zone (mm/mg sample).

Compd.	Species tested		
	*E. coli* (ATCC-25922)	*B. subtilis* (ATCC-6633)	*C. albicans* (ATCC-10231)
**Control (DMSO)**	-	-	-
**2a**	-	13	-
**2b**	-	-	-
**2c**	-	-	-
**3a**	8	-	-
**3b**	-	8	-
**3c**	9	10	10
**3d**	-	-	-
**3e**	-	-	-
**4a**	13	20	-
**4b**	14	22	-
**5a**	-	-	-
**5b**	-	-	-
**Ampicillin**	11	18	-
**Nystatin**	-	-	12

-: No activity, DMSO: dimethylsulfoxide (used as solvent)

**Table 2:** % Inhibition of acute inflammation.

Compd.	% inhibition of acute inflammation			
	1 h	2 h	3 h	4 h
2c	8.695652	18.92308	51.85185*	55.55556***
3c	25.21739	27.69231	78.67647**	81.26984***
4b	1.73913	26.92308	68.38235**	77.14286***
5b	19.13043	20.76923	68.38235**	62.85714***
RF	6.086957	45.38462	60.66176**	69.52381***

Rf: Ibuprofen; Using ANOVA method, at 3H and 4 H all compounds are statistically significant from control * P < 0.05, ** P < 0.01, *** P < 0.001.

**Table 3:** Physical properties and elemental analyses of the new compounds.

Compd.	Yield % m.p.°C	Formula (m w)	Analysis % Calc. (Found)		
			C	H	N
**2a**	85 260	C_12_H_12_N_4_OS (260.32)	55.37 (55.15)	4.65 (4.98)	21.52 (21.32)
**2b**	82 240	C_13_H_14_N_4_OS (274.35)	56.92(56.68)	5.14 (5.37)	20.42 (20.65)
**2c**	86 245	C_13_H_14_N_4_OS (274.35)	56.92 (57.14)	5.14 (5.02)	20.42 (20.64)
**3a**	78 380	C_15_H_10_BrN_5_O_2_S_2_ (436.31)	41.29 (41.05)	2.31 (2.56)	16.05 (16.27)
**3b**	80 310	C_18_H_17_N_5_O_5_S_2_ (447.49)	48.31 (48.65)	3.83 (3.65)	15.65 (15.32)
**3c**	75 360	C_17_H_12_N_6_O_2_S_2_ (396.45)	51.50 (51.27)	3.05 (3.37)	21.20 (21.49)
**3d**	78 370	C_17_H_16_N_6_O_2_S_2_ (400.48)	50.99 (50.67)	4.03 (3.88)	20.98 (20.69)
**3e**	77 330	C_15_H_10_N_6_O_4_S_2_ (402.41)	44.77 (44.53)	2.50 (2.25)	20.88 (20.65)
**4a**	88 278	C_11_H_9_N_3_OS (231.28)	57.13 (57.01)	3.92 (3.76)	18.17 (18.39)
**4b**	81 272	C_13_H_10_N_4_OS (270.31)	57.76 (57.95)	3.73 (3.96)	20.73 (20.50)
**5a**	72 380	C_15_H_11_N_5_O_2_S (325.35)	55.38 (55.29)	3.41 (3.34)	21.53 (21.37)
**5b**	74 270	C_16_H_14_N_6_OS (338.39)	56.79 (56.42)	4.17 (3.98)	24.84 (25.01)

**Table 4:** IR, ^1^H-NMR, and MS of the new compounds.

Compd.	IR (cm^−1^)	^1^H-NMR (δ, ppm) and/or MS
**2a**	3143 (NH), 1663 (C=O), 1240 (C=S).	3.5 [s, 2H, CH_2_(5)], 3.9 [d, 2H, CH_2_(7)], 7.1 (s, 2H, 2NH), 7.3-7.6 (m, 5H, Ar-H), 10.7 [s, 1H, NH(3)]. MS: (m/z) = 260 (M^+^, 6.7 %).
**2b**	3187 (NH), 1671(C=O), 1262 (C=S).	2.1 (s, 3H, CH_3_), 3.5 [s, 2H, CH_2_(5)], 3.8 [s, 2H, CH_2_(7)], 7.0 (s, 2H, 2NH), 7.3-7.5 (m, 4H, Ar-H), 10.9 [s, 1H, NH(3)]. MS: (m/z) = 274 (M^+^, 2.7 %).
**2c**	3187 (NH), 1671 (C=O), 1262 (C=S).	MS: (m/z) = 274 (M^+^, 5 %).
**3a**	3329 (NH), 1650 (C=O), 1234 (C=S).	5.3 [s, 1H, CH(9)], 6.8 [s, 1H, NH(10)], 7.1 (dd, 4H, Ar-H), 11.88-12.11 (s, 4H, 4NH). MS: (m/z) = 436 (M^+^, 10.2 %).
**3b**	3390 (NH), 1660 (C=O), 1235 (C=S).	3.8 (s, 9H, 3OCH_3_), 4.7 [s, 1H, CH(9)], 6.6 [s, 1H, NH(10)], 7.1 (s, 2H, Ar-H), 11.9-12.2 (4H, s, 4NH). MS: (m/z) = 447 (M^+^, 100 %).
**3c**	3225 (NH), 1665 (C=O), 1242(C=S).	4.8 [s, 1H, CH(9)], 6.7 [s, 1H, NH(10)], 7.2-7.5 (m, 5H, Ar- H), 10.9, 11.4, 11.6 (s, 5H, 5NH). MS: (m/z) = 396 (M^+^, 6.5 %).
**3d**	3412 (NH), 1678 (C=O), 1260(C=S).	3.1 (s, 6H, 2CH_3_), 5.1 [s, 1H, CH(9)], 6.5 [s, 1H, NH(10)], 7.1-7.3 (dd, 4H, Ar-H), 11.8-12.0(s, 4H, 4NH). MS: (m/z) = 400 (M^+^, 7.8 %), 385 (M-CH_3_, 100%).
**3e**	3337 (NH), 1675 (C=O), 1338-1541 (NO_2_), 1235 (C=S).	MS: (m/z) = 402 (M^+^, 7.5 %).
**4a**	3386 (NH), 1654 (C=O), 1182 (C=S)	5.2 (s, 1H, H-5 pyrimidine), 6.9–7.1 (m, 5H, Ar-H), 7.8 (s, 1H, N=CH), 11.4-11.8 (s, 2H, 2NH). MS (m/z) = 231 (M+, 22.4%).
**4b**	3360 (NH), 1650 (C=O), 1160 (C=S).	5.2 (s, 1H, H-5 pyrimidine), 7.6-8.2 (m, 5H, Ar-H), 7.7 (s, 1H, N=CH), 11.2, 11.4, 11.8 (s, 3H, 3NH). MS (m/z) = 270 (M+, 16%).
**5a**	3324-3424 (NH_2_), 2225 (CN),1680 (C=O), 1169 (C=S).	3.81 (s, 3H, OCH_3_), 6.9-7.3(dd, 4H, Ar-H), 7.6 (s, 2H, NH_2_), 12.07-12.7 (s, 2H, 2NH).
**5b**	3333-3422 (NH_2_), 2214 (CN), 1662 (C=O), 1197 (C=S).	3.1 (s, 6H, 2CH_3_), 7.1-7.3 (dd, 4H, Ar-H), 7.5 (s, 2H, NH_2_), 12.2-12.6 (s, 2H, 2NH). MS (m/z) =338 (M+, 31%).

## References

[1] Wyrzykiewicz E., Bartkowiak G., Kedzia B. (1993). Synthesis and anti-microbial properties of S-substituted derivatives of 2-thiouracil. Farmaco. Poland.

[2] Sharma P., Rane N., Gurram V.K. (2004). Synthesis and QSAR studies of pyrimido[4,5-d]pyrimidine-2,5-dione derivatives as potential antimicrobial agents. Bioorg. Med. Chem. Lett..

[3] Elkholy Y.M., Morsy M.A. (2006). Facile Synthesis of 5, 6, 7, 8-Tetrahydropyrimido[4,5-b]-quinoline Derivatives. Molecules.

[4] Holla B.S., Mahalinga M., Karthikeyan M.S., Akberali P.M., Shetty N.S. (2006). Synthesis of some novel pyrazolo[3,4-d]pyrimidine derivatives as potential antimicrobial agents. Bioorg. Med. Chem..

[5] Ingarsal N., Saravanan G., Amutha P., Nagarajan S. (2007). Synthesis, *in vitro* antibacterial and antifungal evaluations of 2-amino-4-(1-naphthyl)-6-arylpyrimidines. Eur. J. Med. Chem..

[6] Zhao X.-L., Zhao Y.-F., Guo S.-C., Song H.-S., Wang D., Gong P. (2007). Synthesis and anti-tumour activities of novel [1,2,4]triazolo[1,5-a]pyrimidines. Molecules.

[7] Cordeu L., Cubedo E., Bandrés E., Rebollo A., Sáenz X., Chozas H., Domínguez M.V., Echeverría M., Mendivil B., Sanmartin C., Palop J.A., Font M., García-Foncillas J. (2007). Biological profile of new apoptotic agents based on 2,4,-pyrido[2,3-d]pyrimidine derivatives. Bioorg. Med. Chem..

[8] Sondhi S.M., Singh N., Johar M., Kumar A. (2005). Synthesis, anti-inflammatory and analgesic activities evaluation of some mono, bi, tricyclic pyrimidine derivatives. Bioorg. Med. Chem..

[9] Amin K.M., Hanna M.M., Abo-Youssef H.E., George R.F. (2009). Synthesis, analgesic and anti-inflammatory activities of some bi-, tri- and tetracyclic condensed pyrimidines. Eur. J. Med. Chem..

[10] Khalifa N.M., Ismail N.S., Abdulla M.M. (2005). Synthesis and reactions of fused cyanopyrimidine derivatives and related glycosides as antianginal and cardio protective agents. Egypt. Pharm. J..

[11] Youssif S. (2004). 6-aminouracil as precursors for the synthesis of fused di- and tricyclic pyrimidines. J. Chem. Res..

[12] Mohamed N.R., El-Saidi M.M.T., Ali Y.M., Elnagdi M.H. (2007). Utility of 6-amino-2-thiouracil as precursor for the synthesis of bioactive pyrimidine derivatives. Bioorg. Med. Chem..

[13] Youssif S., El-Bahaie S., Nabih E. (1999). A facile one-pot synthesis of pyrido[2,3-d]-pyrimidines and pyrido[2,3-d: 6,5-d’]dipyrimidines. J. Chem. Res..

[14] Bauer A.W., Kirby W.M.M., Sherris J.C., Turck M. (1966). Antibiotic susceptibility testing by a standardized single disk method. Amer. J. Clin. Pathol..

[15] Harrak Y., Rosell G., Daidone G., Plescia S., Schillaci D., Pujol M.D. (2007). Synthesis and biological activity of new anti-inflammatory compounds containing the 1,4-benzodioxine and/or pyrrole system. Bioorg. Med. Chem..

[16] Winter C.A., Risley E.A., Nuss G.W. (1962). Carrageenin-induced oedema in hind paw of rat as the assay for antiinflammatory drugs. Proc. Soc. Exp. Biol. NY.

